# A Qualitative Study of Participant Feedback on an Acceptance and Commitment Therapy Group-Based Intervention for Parents of Youth with Anxiety Disorders

**DOI:** 10.3390/children13060837

**Published:** 2026-06-21

**Authors:** Jacquelyn Raftery-Helmer, Ashley S. Hart, Alyssa L. Faro, Diana Baez, Phoebe Moore

**Affiliations:** 1 Psychology Department, Worcester State University, 486 Chandler Street, Worcester, MA 01602, USA; dbaez@worcester.edu; 2 Department of Psychiatry, University of Massachusetts Chan Medical School, 55 Lake Avenue North, Worcester, MA 01655, USA; ashley.hart@umassmed.edu (A.S.H.); phoebe.moore@umassmed.edu (P.M.); 3 Department of Psychiatry, Harvard Medical School, 25 Shattuck Street, Boston, MA 02115, USA; alfaro@mgb.org

**Keywords:** child anxiety, acceptance and commitment therapy, parenting

## Abstract

**Background/Objectives:** Incorporating parent training into cognitive-behavioral therapy for anxious youth has not been shown to significantly improve outcomes perhaps because these interventions have not addressed potential interfering psychological barriers to implementing parenting changes and rarely offer between-session support. There is growing evidence that Acceptance and Commitment Therapy (ACT) can target these psychological barriers and generate more flexible and adaptive behavioral repertoires in parents of children with a variety of presenting challenges. **Methods:** Following a pilot trial of “Acceptance and Commitment Therapy for Parents of Anxious Children (ACT-PAC)” a six-week group-based intervention focused on targeting psychological barriers to parenting change using mindfulness and acceptance approaches, we collected qualitative feedback from participants in two post-treatment phases by conducting individual interviews and a focus group with participants that completed the intervention. **Results:** Analysis of interview responses revealed that parents found ACT principles and processes to be helpful, and many also appreciated the ACT-PAC group setting that allowed parents to recognize their experiences were shared by others and to self-disclose in a non-judgmental space. Feedback from the focus group further provides preliminary evidence that ACT-PAC is acceptable to and feasible for parent participants and suggests modifications such as involving additional caregivers, making resources more readily available, and creative structural changes that may facilitate between-session practice. **Conclusions**: Results suggest that the group-based intervention can be both maintained and improved for future participants. Limitations to generalizability in light of possible selection bias and the small focus group sample size are addressed.

## 1. Introduction

Anxiety disorders affect up to 20% of youth [1] and render these children at increased risk for other mental health problems [2]. Although cognitive-behavioral therapy (CBT) protocols are a highly effective, front-line treatment for pediatric anxiety disorders [3,4], up to 40% of children who participate in these interventions show minimal clinical improvement [5,6]. Researchers seeking to improve outcomes of CBT for anxious youth have looked to parenting behavior, such as intrusive or controlling parenting and family accommodation (adjustments made to routines or actions to ease the child’s anxiety), as potential targets for optimization given that these caregiver variables have been shown to contribute to children’s anxiety symptoms [7,8]. However, the integration of parent involvement and training into child-focused CBT protocols for anxious youth has not been found to enhance the short-term effectiveness of such interventions [9,10].

Although most CBT protocols for child anxiety disorders and OCD involve parents adjunctively, some empirically supported interventions incorporate parents as primary participants. For example, family-based treatment for young children with OCD [11] focuses heavily on shaping parent behavior, and Supportive Parenting for Anxious Childhood Emotions (SPACE) [12] is a parent-based program that exclusively targets family accommodation without any direct intervention with the anxious child. In these protocols, changing parent behavior is intended to support child-focused treatment goals, and reduction in child anxiety symptoms is considered the primary treatment outcome variable. However, research indicates that psychological well-being and emotional coping styles in parents are linked to anxiety symptoms in youth, aligning with the broader empirical literature on parental mental health as an important variable that predicts youth mental health outcomes and that is worthy of being its own clinical target (e.g., Beardslee and colleagues [13]).

Parent internalizing symptoms, including anxiety, is associated with greater youth anxiety across multiple studies [14,15,16]. Kerns and colleagues [17] found that mothers’ anxiety predicted ineffective maternal emotion regulation during episodes of child distress, which in turn predicted greater maternal accommodation of child anxiety symptoms, and which in turn predicted higher child anxiety. Both mothers and fathers of young children with OCD also demonstrate difficulty tolerating emotional distress, and fathers’ level of distress tolerance and changes in fathers’ and children’s distress tolerance predicted improvement in OCD symptoms in family-based CBT for pediatric OCD [18]. Notably, the impact of maternal anxiety on child anxiety has been shown to increase with child age [19], a finding that is at odds with the common practice of fading out parental involvement in CBT treatment for anxiety disorders and OCD as children move into adolescence and young adulthood.

Research indicates that the impact of parent psychological factors and behavior on youth anxiety and OCD symptoms is not unidirectional, but rather that these parent–child variables mutually affect each other. For young children between the ages of four and seven with elevated levels of anxiety, the indirect effects of children’s anxiety symptoms on their level of functional impairment were mediated through both parent anxiety and parental self-efficacy [20]. Similarly, a prospective study found reciprocal associations between parent behavior and child anxiety in early childhood (preschool to first grade), such that parent behavior was associated with subsequent levels of child anxiety, and child anxiety predicted increased hostility, decreased autonomy-granting behaviors, and decreased support from parents [21]. These findings point to a parent–child feedback loop in which parental psychological factors and behavior toward the anxious child both contribute to and are influenced by anxiety disorder symptoms in youth.

Despite evidence that implicates parent psychological variables in pediatric anxiety disorders, parent-focused interventions have generally targeted only surface-level change in how parents behave toward their anxious children. They typically teach parents *what* behavior to change, such as coaching parents to be more autonomy supportive, reduce accommodation behavior, and provide reinforcement when their child faces anxiety-provoking situations [22,23,24]. By trying to alter the topography of parent behavior, these interventions neglect to address covert psychological processes that underlie overt parental actions and the reasons *why* parents engage in unhelpful responses. In addition to this limitation, parent-focused interventions for child anxiety disorders and OCD are also typically provided in an individual treatment context—that of the child’s therapy. As an alternative format, group treatment offers the opportunity to focus solely on parent experiences and needs, rather than on the clinical needs of both the parent and child together. It also allows for group cohesion and shared accountability, which have been found to increase engagement in between-session therapeutic homework and skill practice [25].

A clinical approach that addresses some of the limitations of current parent-focused interventions is Acceptance and Commitment Therapy (ACT), an evidence-based psychotherapeutic modality that aims to increase psychological flexibility and values-congruent behavior [26]. The ACT model consists of six interconnected core processes that can each range from more to less flexible; psychological inflexibility is represented by (1) lack of contact with the present moment (mental attention being stuck in conceptualized past and/or future), (2) cognitive fusion (relating to thoughts as literal content), (3) attachment to the conceptualized self (a sense of identity based on evaluations and stories about the self), (4) experiential avoidance (difficulty tolerating uncomfortable inner experiences), (5) lack of values clarity, and (6) patterns of behavior that involve inaction, impulsivity, or avoidance. Research highlights the relevance of these processes to families of youth with anxiety disorders. Feinberg and colleagues [27] reported that higher degrees of parents’ negative beliefs about their child’s anxiety (e.g., “My child can’t tolerate being anxious” and “If my child gets too anxious, it could be harmful”) and parental experiential avoidance (e.g., a parent’s unwillingness to experience and attempts to control their feelings of distress in response to their child’s anxiety) are associated with greater family accommodation. Parental experiential avoidance is also associated with higher levels of parental control [28].

There is growing evidence that ACT and other treatments that integrate mindfulness and acceptance practices [29] can generate more flexible and adaptive behavioral repertoires in parents of children with a variety of presenting challenges [30], including children with autism [31,32], oppositional behavior [33], chronic health conditions [34,35,36], and separation anxiety [37]. In many of the clinical trials examining the efficacy of ACT for parents, the intervention has been delivered in a group format, allowing parents to have a time and space dedicated to developing psychological flexibility skills separate from their child’s individual treatment and to receive support from other participants who experience similar struggles in relation to their children.

Regarding its clinical application to parents of youth with anxiety disorders, ACT can help these caregivers learn to bring their attention more readily and consistently to the present moment (through mindfulness training), create mental distance and view their identities as separate from unhelpful or inaccurate thoughts about their child’s anxiety (by engaging in cognitive defusion and perspective-taking exercises), foster willingness to experience their own emotional distress (through distress tolerance and acceptance-based practices), hone in on what is important to them about how they want to parent (through values clarification exercises), and take specific steps that align with their identified parent values (by practicing committed action) so they can respond more intentionally and effectively in the face of their child’s anxiety rather than resort reflexively to avoidance, hostility, overcontrol, or accommodation [33]. In this way, parents can shift from anxiety-driven reactions to values-consistent parenting responses that promote approach rather than avoidance behavior in their anxious child, facilitating the child’s long-term growth, independence, and resilience.

Given the limitations of parent-focused interventions for pediatric anxiety disorders, the theoretical relevance of ACT for addressing the underlying origins of behavior that characterizes parents of anxious youth, and data indicating that ACT can improve the psychological flexibility and wellbeing of parents of children with mental health challenges [31,38,39], we previously conducted an open-trial pilot study to test a group-delivered ACT intervention tailored for parents of children with anxiety disorders (ACT-PAC [40]). We were equally interested in and measured mental health outcomes for both parent participants and their children with anxiety disorders, who did not receive any direct intervention as part of the study. Data from 23 parent–child dyads revealed that following the six-week ACT-PAC group protocol, primary caregivers of youth with anxiety disorders demonstrated slight decreases in self-reported levels of cognitive fusion, and children were rated by their parents as having fewer internalizing symptoms [41]. Overall, parent participants reported a high level of satisfaction with ACT-PAC on the Client Satisfaction Questionnaire that was administered at the post-treatment assessment.

While these initial findings are promising, they do not adequately speak to the acceptability or feasibility of ACT-PAC as experienced by parent participants, which is particularly important to evaluate in light of research showing that families are more likely to drop out of parent-only interventions compared to traditional CBT [42]. Within the last several decades, there has been a call for the use of more qualitative research that may offer a deeper understanding of how patients experience the delivery of mental health services than quantitative methods may be able to do alone [43]. Such methods may aid in the interpretation of the results of quantitative studies or further contextualize findings [44,45] and they offer a platform for participants’ voices to be represented [46]. Based on feedback solicited from participants in the ACT-PAC intervention, protocol modifications can be targeted to the experiences and preferences of parents to enhance the clinical impact and external validity of the treatment. Qualitative data from participant feedback can also aid in ascertaining whether and how to proceed with larger-scale empirical evaluation (e.g., randomized clinical trial), implementation, and dissemination of ACT-PAC as an intervention that can enhance treatment outcomes for youth with anxiety disorders.

The aim of the current study was to analyze qualitative feedback obtained at two post-treatment phases, in both an individual and group format, from parents that participated in ACT-PAC groups from the open-trial pilot intervention study [41]. Specifically, we sought feedback on (1) what participants perceived to be the helpful features of the intervention, (2) barriers they experienced in generalizing newly learned skills, and (3) general recommendations for implementing and disseminating the ACT-PAC intervention. Both focus groups and individual interviews were included as several researchers in the healthcare space [47] have found that the combination of individual interviews and focus groups can offer a more nuanced understanding of clinical phenomena. In our particular context, we believe that data triangulation through the integration of multiple qualitative methods would allow for a more enhanced understanding of participants’ experience of ACT-PAC. The individual interview data would allow us to gather general impressions of the intervention and the focus groups that followed, would support more detailed descriptions of how participants experienced the intervention, further enriching our understanding of both the acceptability and potential limitations of ACT-PAC. Thus, the separate date sources were expected to be mutually informative. This design also allowed for convergence across focus groups and individual interviews, enhancing the trustworthiness of study findings.

A priori codes for qualitative analyses were first developed by the research team, consistent with a framework analysis approach [48]. Codes were analyzed using an “immersion/crystallization” approach [49], in which the data were organized and then crystalized into the most important topic. Qualitative data will inform modifications to ACT-PAC and plans for conducting a randomized controlled trial of a revised intervention protocol that has been enhanced by participant input.

## 2. Study 1 Method

### 2.1. Study 1 Participants

Participants in the ACT-PAC open-trial pilot study were primary caregivers (20 mothers, 3 fathers, mean age = 45 years) of twenty-three youth ages 7–17 years (14 males, 9 females; mean age = 12.7) who met criteria for a primary anxiety disorder based on the Anxiety Disorders Interview Schedule for Diagnostic and Statistical Manual of Mental Disorders, 4th Edition (DSM-IV): Child and Parent version [50] at the pre-treatment assessment. All participants identified as White, and parents were highly educated, with 17% completing some college, 48% completing college, and 26% reporting post college education (education was not reported for 2 participants). Four groups of up to 5 to 7 parents participated in the ACT-PAC group intervention. For additional information about participants, recruitment, and methods from the original ACT-PAC pilot study, see Raftery-Helmer and colleagues [41].

Qualitative feedback was obtained from all parents that participated in ACT-PAC treatment groups (*n* = 23) via individual interviews at the post-treatment assessment that were conducted after the six-week group intervention. Additional information about ACT-PAC [40], including a session-by session outline, can be found in the original ACT-PAC pilot study [41].

### 2.2. Study 1 Interview

The Qualitative Feedback Interview, designed specifically for this study, consisted of three open-ended questions and was administered by a trained research assistant to each ACT-PAC participant individually as part of the post-treatment assessment battery. The interview asked all 23 participants about (1) their overall experience with the treatment study; (2) the most and least helpful aspects of the group treatment program and suggested improvements; and (3) any additional feedback participants had about the ACT-PAC intervention or study (see Appendix A for specific questions).

### 2.3. Study 1 Analytic Plan

Qualitative interviews were analyzed using a methodology referred to as “coding, consensus, co-occurrence, and comparison [51] that used an iterative approach rooted in grounded theory [51]. Segments of text (phrases to several paragraphs) were assigned codes using an a priori codebook that was developed by project investigators based on the interview guide and emergent themes. The final list of codes was constructed through consensus of research team members and consisted of a numbered list of themes related to participants’ overall impressions of ACT-PAC (e.g., were participants’ experience of ACT-PAC favorable, would they recommend the treatment, what were the most helpful and least helpful features of the treatment group, what were suggested improvements, what were barriers to treatment engagement). The interview was then independently coded by two trained research assistants using this final codebook, who both met regularly with each other and the first author to discuss progress and resolve coding discrepancies. “Coding by consensus” in this way by regularly meeting to discuss procedures for assigning codes to segments of data and resolving differences in coding procedures is most consistently used as a measure of reliability in qualitative data analysis in the mental health services area [52,53,54]. The resulting codes were analyzed using an immersion/crystallization approach [49]. After coding the qualitative texts line-by-line, the codings were then ordered into discrete themes using pattern level analyses [51]. Finally, in step 3, by constantly comparing the categories with each other, the discrete themes were organized into higher order patterns. After identifying discrete themes and higher order factors, we then applied an enumerative approach [55] to explore the salience of these themes by investigating the frequencies of the predetermined codes. We calculated the frequencies of the themes that emerged from the immersion/crystallization approach.

### 2.4. Study 1 Results

#### 2.4.1. Favorable Treatment Impression

Qualitative analyses of post-treatment individual interview responses yielded a variety of higher order factors related to participants’ overall experience of the group treatment program and suggested improvements. One prominent higher order factor identified was overall impression of the group treatment program, which included the discrete theme of favorable impression. Eighty-four percent of participants expressed favorable impressions of the ACT-PAC group treatment. One participant described her positive experience of the intervention in this way: “The value of the group itself was wonderful. It gave insight, perspective, a feeling of not being alone, and shared strategies for parenting. I’d be interested in continuing with group work.”

#### 2.4.2. Preferred Treatment Features

Study participants also identified aspects of the group treatment program they found to be most helpful. Participants (*n* = 19; see Table 1) frequently referred to nonspecific features of the intervention, including opportunities to receive peer support and a non-judgmental space for self-disclosure. For example, one participant described the group as a Nonjudgmental place. Found it very comforting with the other mothers in the group that I was not alone—some of the feelings you feel with children is hard, having feelings and thoughts that make you feel worse about yourself. Comforting to know that I was not alone, and that didn’t make me a bad parent.

Another participant described ACT-PAC as a “safe space to voice my concern.”

Multiple participants (*n* = 8) reported that ACT-specific features of the intervention were the most helpful, pointing to possible intervention-specific mechanisms of change. Analyses identified discrete themes of mindfulness practice and decreased parenting reactivity (*n* = 5). This is illustrated by one participant’s statement:

Had heard of mindfulness before but not in that context, not just with breathing but with thoughts. Helped in my parenting all around. Have been eating better and trying to exercise and trying to choose things for myself whereas I wasn’t before. Trying to keep it in my mind and keep the papers out and not fall into those habits I’ve been doing forever.

For another participant, the most helpful aspect was “learning to focus more on my reactions and to see how my responses feed into tension in the house.”

### 2.5. Unexpected Focus

The most commonly identified area that would benefit from clarity was the unexpected focus of ACT-PAC being primarily on parents’ experiences and behaviors and not on those of their anxious children. As one participant noted:

It was not clear to me what the parenting group would “teach.” I came into it thinking I would learn strategies to parenting my anxious child. While that was somewhat true, I primarily learned strategies for mindfulness with my own thoughts and emotions, including how they relate to parenting.

While some participants (*n* = 5) described this focus on the parent experience to be a shortcoming of the intervention program, others (*n* = 8) provided detailed descriptions of how this focus, although unexpected, turned out to be helpful. One participant described her perception that the focus on the parent experience was more helpful than an intervention addressing her child’s anxiety alone:

Honestly, I had no thoughts on the improvement part of it—not what I expected at all, thought it was going to be more about how to deal with my child and what she was struggling with as opposed to helping me concentrate on myself, and it was more helpful.

### 2.6. Suggested Improvements

A feedback theme related to suggested improvements to ACT-PAC involved ways to enhance treatment engagement outside of the group. Several participants (*n* = 3) recommended increasing accountability from the group for between-session practice. One participant explained, “I think there would be greater self-accountability for completing daily exercises/tasks during the week.”

Participants (*n* = 3) also suggested the group treatment could provide additional resources, particularly opportunities to continue or extend engagement in skills practice or a group-based intervention. For example, one participant stated, “I think it would have also been nice to receive a resource list at the end with either recommended materials and/or possible area groups that we could look into joining if we found the group to be helpful.”

A third suggestion for improving treatment engagement related to the structure of the group sessions. Participants (*n* = 8) noted that having the group run over a longer duration could help to solidify and maintain skill acquisition. For some (*n* = 3), the 6-week treatment was perceived as too brief to learn skills, incorporate them into daily practice with their children, and receive support for inevitable challenges that occur with more sustained behavioral changes. According to one of the participants, “I think the group cut off too soon for long-term results. I certainly would have benefited from more reinforcement check-ins, even if they were just a couple of shorter sessions spaced further apart at the end.”

## 3. Study 2 Method

### 3.1. Study 2 Participants

Qualitative feedback was also obtained from parents that participated via one in-person, single-session, 90-min focus group conducted at least 12 months after completion of the ACT-PAC groups. All participants from the ACT-PAC open-trial pilot study were invited to participate in the Study 2 focus groups; in response, 7 of the 23 parents from the ACT-PAC pilot study agreed to participate following invitations via email and telephone call.

### 3.2. Study 2 Focus Group

The focus group consisted of semi-structured questions that were directed at gathering information about what participants liked best and least about the intervention, their experience practicing and generalizing skills outside of group sessions, maintenance of skills following the completion of the group, and overall suggestions (see Appendix A for sample focus group questions). The focus group session was videotaped, and video data was transcribed before coding. The transcriptions were checked and reviewed for accuracy by two research assistants.

Using a similar analytic plan to phase 1, two coders independently completed two stages of analyses. First, we used an immersion/crystallization approach [49], coding the transcribed qualitative data line-by-line. Any disagreements in assigned codes were resolved through discussion between coders and enhanced definition of codes. These codes were ordered into discrete themes using pattern level analyses [51] and then organized into higher order patterns using QSR NVIVO [56].

Lastly, we used an enumerative approach [55] and examined the frequencies of coded text to describe the relevant salience of themes that had emerged from the immersion/crystallization approach.

### 3.3. Study 2 Results

#### 3.3.1. Positive Features

Participants in the focus group also described aspects of the ACT-PAC intervention that they found useful or helpful. The three positive features that emerged included social support and the two ACT processes of mindfulness and cognitive defusion.

#### 3.3.2. Non-Specific Feature: Group Support and Sharing Common Experiences

Multiple parents (*n* = 4) reported that the group intervention offered an opportunity to connect to other caregivers struggling with similar parenting challenges, and that it ameliorated the sense of loneliness that they often experienced while parenting an anxious child. As illustrated by one participant, “We all had the same story, like you didn’t feel alone.” Similarly, other participants added,

…the support aspect, to know that we weren’t alone and to hear how others were handling sort of, um, their issues. But I think th-the part that was best for me was feeling relief in some ways, not that I’m glad you guys are all doing this. You know that, others are going through this, and handling it, and, um, the support aspect of it.

Yeah, it definitely was nice to hear that other people were struggling as well as having successes, cuz it-it definitely balanced the like, ya know, those weeks when it felt like all we did was struggle versus those weeks where you’re like ya know “Hey this did work.” and you know you can bring each other up.

These statements highlight the comfort parents reported experiencing from learning that others shared their parenting struggles, a known non-specific benefit of group therapy [57].

### 3.4. ACT-Specific Content

Throughout the focus group, participants identified several specific skills and practices completed during the group sessions that they liked the most or practiced. Although their responses did not necessarily provide information about the durability of these skills, or the extent to which they were maintained by participants at 12-months follow-up (i.e., support for efficacy) they did provide direct support for which skills and exercises were acceptable to participants and feasible to implement. Specific support for the acceptability of content focused on mindfulness and cognitive defusion is elaborated below.

### 3.5. ACT-Specific Content: Mindfulness

Mindfulness, the practice of bringing one’s attention to the present moment while foregoing judgment about what is presently occurring, was reported as among one of the more helpful features of the intervention and the most practiced skills outside of treatment (*n* = 5). Participants noted that because mindfulness was one of the first skills introduced in the ACT-PAC group intervention, they were afforded more time and opportunities to practice it. As described by one parent:

Probably the mindfulness one for me… one of the beginning ones and I had the whole six weeks of constantly doing the mindfulness so that one kind of stuck after a while. I know-probably think for weeks four and five I don’t remember much, I think probably, I remember putting things into categories, but I don’t remember much after that. But the whole just, trying to be aware of what’s going on now, is probably the most important one for me, but it’s also probably the most important because it was like one of the first ones we did, and that one sunk in more.

Another parent said that mindfulness, although not necessarily their favorite skill, was the most memorable technique, which may speak to the generalization of the skill outside of intervention sessions. As stated by one parent:

Yeah, I don’t know if I liked it the best, but the one I remember the most, and probably use the most now would be the mindfulness. And I don’t know if it was just—just the skill that I understood the most.

Other parents said that they practiced the skill of mindfulness regularly and implemented it during interactions with their own child. This parent stated:

Yeah, and I think mine too which is why I have “Insight Timer” and “Mindful” on my phone. I use them every day, um, since then, I really enjoy it and of course [CHILD NAME] hates that I make her do it with me now.

### 3.6. ACT-Specific Content: Cognitive Defusion

Cognitive defusion, the practice of observing thoughts and other cognitions as temporary mental experiences without getting caught up in their content, was reported as one of the most utilized skills and the easiest to generalize outside of session. Parents (*n* = 4) identified cognitive defusion as a helpful skill and reported regularly integrating cognitive defusion practice into their daily lives. This was illustrated by the following statement:

I think the defusion definitely was, really became part of our life. Uh, mindfulness was really great in the beginning and we kind a had fallen off. And I think there is much more awareness now of, for me, when I am behaving in those ways that are counterproductive. And just that alone it—I take a step back from the situation and I think it’s generally helpful, but probably could do better.

Another parent expressed that this technique helped in response to stress, and one that they used in other aspects of their life, including at work. That parent noted:

And I think the defusion is definitely something that I use a lot daily. And even in my own work, I work as a therapist, um, in another setting and I’ve learned that taking a step back and just when somebody is having a meltdown when somebody is having a problem or even if it’s at home.

### 3.7. Barrier to Generalizing Skills: Limited Accountability Structure

Analysis of the focus group transcript identified three primary barriers to generalizing skills: limited accountability structure for between session practice, co-parenting challenges, and access to content between sessions.

Several parents (*n* = 4) reported they struggled to prioritize using newly learned skills at home or outside of the weekly sessions, noting that an accountability structure that incentivized practice was not built into the treatment program. While at-home practice activities were assigned weekly, parents noted the absence of regular monitoring or feedback provided on participants’ engagement in these exercises between ACT-PAC sessions. This was illustrated by the following statement:

That’s why I definitely agree with the accountability and almost like a guided practice where you-where it was something new that we were all trying to do where we could, like, set aside the time or if we had to be accountable everyday then it was, I think it made you a little more aware ‘cause it was, like homelife goes by and you’re like “Oh my gosh, a week has gone by and I didn’t do it.

Although parents reported finding ACT-PAC skills helpful, they generally reported that they were not remembering to actively apply the strategies and techniques learned in sessions to their everyday parenting situations at home, likely undermining the benefits that the treatment could offer. As described by one parent,

The barrier for me, it was, um, prioritizing and being accountable. So, uh, you know sometimes a day would go by and I didn’t have to do it that day and I’m like ‘Oh gosh I-you know-I’ll get to it tomorrow’ even though I wanted to do it sometimes I just didn’t make a priority so I-accountability sort of, which again is on us, but if it can be done that we’re forced to be accountable it’ll happen and that’ll benefit all of us.

Participants put forth several potential solutions to address the challenge of between-session skill practice. For one parent, meeting twice per week could offer an accountability structure for parents to practice and use newly learned skills more frequently. This parent stated, “That’s a good point ‘cause if you have the second session in the same week you can, then it’s just my accountability part ‘cause it’s like ‘Oh yeah, I’m supposed to be’.” For other parents, self-generated accountability, in the form of between-session reflective activities, had the potential to offer the kind of structure to help parents more consistently apply learned skills to actual interactions with their children. For example, one parent noted,

I think that accountability was big. And it also kind of helps you reflect back, like writing down what you did, when you did it, and kind of what your thoughts were was good for accountability but then it also helped me at least, to kind of think back and assimilate, and see progress I guess.

Despite parents reporting that the skills that they learned were helpful, many attributed the struggle to apply or remember to apply the skills to actual interactions with their children to the lack of built-in accountability in the ACT-PAC treatment program. They operationalized this built-in accountability as more frequent check-ins with parent participants by group facilitators, or mechanisms by which parents could monitor their own progress in utilizing new skills, such as by using monitoring forms to, as the above participant noted, “writ[e] down what you did, when you did it, and kind of what your thoughts were.”

### 3.8. Barrier to Generalizing Skills: Co-Parenting Challenges

Another identified barrier to generalizing ACT-PAC skills outside of weekly group sessions was the challenge of implementing the skills alongside a co-parent who did not attend the treatment sessions. Several parents (*n* = 5) reported arguing with their partners about how and when to implement their newly learned skills, especially when their co-parents did not understand the goals of these new approaches or how to implement them with the child. As a result, some parents reported perceiving that they were less aligned with their co-parents on how to approach their child’s anxiety from an ACT-based lens and that they struggled to convey or teach ACT principles to their co-parent. This was particularly illustrated by the following statement:

Cause I agree it was really hard to tell my husband, like what it was that we were doing, and then I did find there were a couple arguments between he and I where I’d be like, he’d be doing the old stuff and I’m like “No we’re not supposed to do it like—this is not how we handle this anymore.” It’s just really hard to explain that and, you know, then I kind of felt like he stepped back, like wasn’t gonna parent anymore. There was some of that, until you could kind of, like, figure it out.

Other parents reported attempting to explain the newly learned skills to their partner, but often feeling that they were unable to do so effectively. This unequal knowledge of the ACT-PAC approach and techniques was perceived as potentially undermining the effectiveness of the intervention. As described by one parent:

One thing that I—that hasn’t been really mentioned that kind of was a barrier was um, just only having one parent. And going home and then trying to explain it to the other parent involved and sort of probably really not doing it justice, and it’s really—it’s much easier to have two people in the same household on the same page. And otherwise, its—you know I’m trying to preach to them this is how we should all be behaving and, you know, it’s easier when everybody is you know, on the same page.

Several potential solutions were proposed by parents to address this barrier. A few parents thought that a recap session at the beginning or end of the six-week group program could be helpful so that co-parents could be exposed to, at a high level, the skills taught in session; they anticipated that this broad overview for co-parents would increase their willingness and ability to participate and collaborate in the implementation of ACT-PAC skills with their child. As stated by one parent,

Yeah, and I mean even if it’s not realistic ‘cause I was gonna say, if a parent has to stay home with the child, like even if there was like one session either in the beginning or the end where the second parent came to kind of get a summary of the process not just from us, like from a neutral third party, or a video recap.

Other parents thought that more in-depth exposure to ACT-PAC content, such as a video recording of the session, would be more helpful. One parent commented,

Like some classes, the hol—they can do, I—I don’t know I haven’t done it, but, you know, they—they hold the interaction online or the recap online where you can watch the lecture online. So, go home and say, “Here’s what we did today.” And have them watch the quick clip so that you know what we were taught.

Other parents thought two sessions a week, one for each parent, could be beneficial. This would mean that both parents would receive equivalent content, both in the format of the intervention and content delivered. For example, one parent said:

Yeah, I mean I think it would be difficult to have two parents in one session and relying on having everyone have childcare. A lot of times that’s not a possibility, but, or even if like what we were talking about two sessions if one session is for one parent and one was for the other and ya know, so that way, we are both kind of getting the same presentation.

Despite parents reporting that the content and skills covered in the intervention were useful, they also reported that it was hard to fully benefit from the treatment when their co-parent, for lack of understanding, may have interfered with the implementation of these techniques during in-the-moment interactions with their children. Adding opportunities for both parents to learn the ACT-PAC approach and skills, either through brief dissemination of content, audio recording resources, or simultaneous sessions could result in better generalization of the skills through improving parent alignment and collaboration during interactions with their anxious child.

### 3.9. Barrier to Generalizing Skills: Forgetting and Difficulty Applying Content Between Sessions

A barrier that many parents discussed during the focus group was difficulty remembering the content between sessions, particularly because they lacked access to content material between the sessions. Several of the participants (*n* = 6) reported not using the newly learned skills because they could not remember the content. This was illustrated by the following statement:

I think toward the end it was like there were so many things to remember like week after week after week. It was like there were some weeks I would be able to do like, this and that but not get to the other…you know what I mean? It was sort of, because it built on itself. I think towards the end I definitely was not getting everything like all done.

Many parents reported finding it challenging to keep up with every skill, as they learned additional content week after week, without any at home resources. As described by one parent,

In the first couple of weeks, they’re kinda like getting you going and now in weeks three, four, and five it’s like, okay, now I’m learning all these new things, and I still have to remember weeks one and two.

Participants identified a possible method to address the overall difficulty of remembering content between sessions. One participant described how helpful it would be to consolidate a collection of resources highlighting the content that was learned that session, as a way to remember content and home and aid them in attempting to practice and implement skills between sessions. As proposed by one parent,

Some sort of binder that we have on the shelf that we can pull out and flip through here’s “Gah, that’s right! That’s what we’re supposed to do. That worked so well.” Uh, because in the crux of the moment you tend to lose it.

Another parent suggested providing parents an online link that could serve as an archive of what they learned to look back on outside of sessions. This parent stated,

If there was some link that we could go to that kind of summarizes everything and then even if you could, you know—for me I know I—I get that the groups, that it’s hard to do a weekly thing but I have to say to be able to have it ongoing would just be really huge.

### 3.10. Future Directions: Expanding Target Population

Several participating parents proposed (*n* = 3) that the ACT-PAC intervention would be beneficial for all parents, regardless of whether their children have an anxiety disorder. They noted that ACT-PAC techniques and approaches were helpful for the entire family, and they often found themselves generalizing the content to siblings of the child with the anxiety disorder diagnosis. As illustrated by this parent:

I agree, I—uh—I don’t remember who was saying it, parenting these children, we have to parent them differently but I feel like—I’ve got one child without anxiety and I feel like all of this still helps her tremendously. So, I think it’s helpful just, I don’t even think it relates just to parenting.

Another parent recommended a more inclusive screening process so that a larger group of parents could benefit from the ACT-PAC intervention. This parent stated,

I think the only thing would be like, ya know, I mean obviously this was just a study not just an ongoing, ya know, resource for people. But I think this would be helpful for a lot of people who don’t specifically match the criteria that we had going on here. So, I mean obviously you guys had to screen us in and make sure everybody was, ya know, ya know, have certain qualifications to get into the study, but not having ya know, such stringent qualifications would probably benefit a lot of people.

This feedback underscores the applicability of the ACT-based intervention to the experience of the parenting of children and adolescents. It also suggests the utility of expanding the targeted group served to parents whose children have clinically elevated anxiety symptoms or are at risk for an anxiety disorder and testing ACT-PAC as both a preventive and treatment intervention.

## 4. Discussion

This dual-phase qualitative feedback study solicited ACT-PAC participants’ experiences of the group-based intervention as well as their suggestions for protocol and program modifications. Feedback was collected from parent participants in both an individual interview format at the post-treatment assessment and via a focus group conducted at least a year after ACT-PAC concluded.

The two features of ACT-PAC that parents reported finding most useful—mindfulness and cognitive defusion—are psychological flexibility practices rather than behaviorally focused techniques. This underscores a primary goal and purpose of ACT-PAC: to address the underlying psychological factors that can support and engender behavior change in parents of children with anxiety disorders. Given that a substantial subset of participants identified cognitive defusion as their favorite feature, and results from the ACT-PAC pilot study [41] showing a significant reduction in parents’ self-reported cognitive fusion following the group treatment, future research can examine whether this component drives intervention effects. It may also be clinically useful to increase skill building in this area in the ACT-PAC protocol.

Parents also reported benefitting from the group-afforded opportunity to connect with other caregivers struggling with similar parenting challenges. They noted that this connection ameliorated the loneliness they often experienced while parenting an anxious child. In fact, many parents felt that the six-week treatment duration was too brief because they wanted to continue to receive peer support for inevitable challenges that occur with more sustained behavioral changes. Finding ways to capitalize on this peer support—both between sessions and across the duration of active treatment—may help to maintain long-term treatment gains and enhance parent well-being. It will be important to explore whether positive treatment outcomes were primarily due to the nonspecific positive experience of the group format, which allowed parents to hear and relate to others’ experiences, or whether the outcomes were more attributable to ACT-specific treatment components. If the former is mostly or largely accountable for treatment outcome, it would be useful to consider a less resource-intensive intervention (e.g., support group for parents of anxious youth) as an alternative.

Collectively, participants’ feedback points to a number of recommendations to improve the implementation and impact of ACT-PAC. Participants described challenges generalizing and maintaining skills outside of sessions. There were three broad barriers: limited accountability for between-session practice, difficulty applying skills during spontaneous parenting interactions, and co-parenting challenges.

First, parents identified that the limited accountability structure of the intervention curtailed between-session skill use. While at-home practice activities were assigned weekly as a component of treatment and made available to participants to facilitate between-session practice, parents did not experience them as sufficient for supporting out-of-session practice in the absence of a built-in monitoring system that ensured participants completed activities or that provided structured feedback. While additional tracking or monitoring of at-home activities could increase engagement through external contingencies (e.g., praise for participation), presenting a clearer rationale for between-group practice may increase parents’ autonomous motivation. However, the success of any implemented accountability tool hinges on the extent to which participants can incorporate such systems into their daily parenting life, which likely will vary as a function of the acceptability and ease of using a particular tracking tool. The findings nonetheless underscore the importance of a follow-up check-in structure that encourages between-session skill use.

Parents also reported difficulty generalizing intervention material to their own parenting scenarios, a common criticism of parenting interventions [58]. This concern may reflect the challenges of learning skills outside natural parent–child interactions. Results point to the utility of providing augmented support that is not tied to time or place, which may allow parents of anxious children to practice skills either when they have the opportunity to do so, or when practicing skills is most necessary (i.e., during challenging interactions with their child). Creating an archive of sessions materials or utilizing self-help models, web-based interventions, and video conferencing all represent feasible systems of delivering content that might enhance treatment generalization and skill maintenance.

Another barrier to generalizing ACT-PAC skills was the challenge of implementing the skills alongside a co-parent who did not attend the treatment sessions. Notably, the majority of the parents that participated in ACT-PAC, and all of the participants in the focus group, were mothers. It is worth considering how parent gender may impact engagement in treatment and how to include additional caregivers (in homes where there are multiple caregivers) such that intervention effects are not diluted by competing views at home about how to respond to the child’s anxiety. Several potential solutions were proposed by parents to address this barrier that ranged from recap sessions that would allow for both parents to be exposed to therapeutic content at a high level to opportunities for parents to be delivered equivalent content, with sessions offered twice a week (one for each parent). In sum, future research should evaluate a modified ACT-PAC intervention that incorporates specific recommendations made by participants, such as the inclusion of a co-parent, creation of an archive of session materials, and revised structure that includes a follow-up check in. While ACT-PAC may lead to significant skill-building for parents under ideal circumstances, these effects may be diluted if the features of implementation do not maximize opportunities to access and practice skills between sessions or specifically address barriers to implementing learned techniques with fidelity.

One unexpected finding was that some parents felt that a larger group of parents, not just those whose children meet clinical criteria for child anxiety disorder, could benefit from the ACT-PAC intervention. Such findings suggest that if we were to proceed with larger-scale empirical evaluation (e.g., randomized clinical trial), implementation, and dissemination of ACT-PAC, we may consider expanding it, in a prevention-oriented approach, to anxiety-impacted families more broadly, including families with anxious parents, or children whose presentation is subclinical and are therefore at-risk for the future onset of an anxiety disorder. Future research may also identify treatment moderators by systematically examining whether some families may benefit more from the intervention, such as those in which both the child and parent experience clinically significant anxiety disorder symptoms.

The overall positive response of parents to the ACT-PAC intervention documented here and the encouraging clinical findings reported in the previous study [41] support the use of ACT as an additional parent-focused intervention that can benefit children with anxiety disorders. Clinicians using traditional CBT protocols to individually treat anxious youth can assess for indicators of psychological inflexibility in parents, such as adherence to negative beliefs about their child’s anxiety being harmful (e.g., using the Cognitive Fusion Questionnaire) and difficulty staying present with their child and with giving space to their own emotional distress in relation to their child’s anxiety symptoms (e.g., with the Parental Acceptance and Action Questionnaire [28]). They can incorporate ACT interventions targeting these processes into parent sessions and assign practice exercises to parents, such as mindfulness and values clarification activities, alongside between-session homework tasks for the anxious child. Clinicians may also consider offering an ACT-PAC group to parents to promote systemic, family-based change in psychological flexibility for youth with anxiety disorders. Current data suggest that ACT-PAC can serve as an adjunctive treatment for pediatric anxiety disorders; additional research is needed to more rigorously test the efficacy of the protocol as a potential standalone, exclusively parent-focused intervention.

## 5. Limitations

There are important limitations to this study worth noting. Study participants all identified as White and highly educated, and given that they were recruited from a pediatric OCD and anxiety disorders clinic, had high treatment access. This limits the generalizability of our study findings to more socioeconomically and racially diverse populations; additional research is needed to ascertain whether ACT-PAC would be beneficial and acceptable to more demographically diverse families or whether they would have additional recommendations for intervention improvements. Developing potential cultural adaptations to ACT-PAC, tested on more diverse populations, would enhance the study’s impact, especially in light of research showing that culturally adapted and tailored interventions are more effective than standard interventions [59,60].

The Study 2 sample was also small and consisted of a subset of parents who, when contacted at least one year following their participation in the original ACT-PAC treatment study, agreed to be part of the focus group, thus introducing the possibility of selection bias. It is possible that only the most satisfied participants agreed to participate in the focus group. Nonetheless, this study makes an important contribution by offering a deeper understanding of how participants experience the delivery of mental health services [43] and helping to interpret and contextualize findings from the open trial pilot study of ACT-PAC for parents of children with a primary anxiety disorder or OCD diagnosis [41].

## 6. Future Research

Future research could consider exploring ways that we might re-design ACT-PAC to address some of the current barriers that parents reported with accessing content between sessions and generalizing skills during acute parenting challenges. Innovative technological advances, such as wearable technology, could provide a structure for prompting or reminding parents to check in on their use of the skills or provide in the moment access to material from the group. For instance, parents could be issued smartwatches that could monitor heart rate, and provided prompts to utilize ACT-PAC skills if the heart-rate range indicated physiological reactivity. The feasibility of similar digital augmentations has been established [61] and could be prioritized for future work given noted barriers to routinely utilizing learned skills outside of sessions.

Another possible innovative redesign is to incorporate bug-in-ear technology that allows for parent coaching to occur (discreetly) in real time through the use of a two-way communication headset [62]. This would allow parents to practice newly learned skills with their children and to receive immediate corrective feedback [63,64], instead of receiving feedback a week later based on a retrospective report of how they had applied ACT skills. There is a well-established evidence base of the success of bug-in-ear training as part of PCIT [65]. Future iterations of ACT-PAC may consider an additional intervention component of having parent dyads meet together to complete behavioral tasks designed to elicit child distress and parent reactivity (e.g., conflict conversation, conversation about something the child is anxious about). The therapist could observe these interactions, and, via bug-in-ear technology, provide in-the-moment, ACT-consistent feedback at a time when parents are exhibiting unhelpful responses or reactivity, as a way to help parents better access and apply new skills.

In addition, there are emerging conversations about using generative AI tools in the parenting sphere. As one example, the parenting influencer “Dr. Becky” recently released an app that allows parents to bring parenting challenges to an AI chatbot, which has more than 900,000 paying members [66]; this points to a demand for using technology to support parents during acute parenting challenges. Offering a similar tool trained specifically in ACT-PAC content could provide parents with immediate feedback during moments with their child when ACT skills may be most beneficial but also difficult to access without additional support. While these three innovative ACT-PAC redesigns may be worth exploring, they all raise further questions about their acceptability, feasibility, and general concerns related to privacy that exist particularly when introducing generative AI technology.

## 7. Summary

Building on a pilot study that yielded quantitative data supporting the efficacy of ACT-PAC as a caregiver-focused intervention for pediatric anxiety disorders [41], qualitative feedback gathered at two time points and via two methods indicates that aspects of the group-based intervention can be both maintained and improved for future participants. A qualitative feedback-enhanced ACT-PAC protocol can be tested in future research, with the ultimate aim of improving outcomes for youth with anxiety disorders.

## Figures and Tables

**Table 1:** Frequency of caregiver responses to individual interview and focus groups.

	**Individual Interview Feedback**		
Higher order code	Code	Definition	Number of Participating Caregivers That Reported Code (*n* = 23)
Favorable intervention impression		Positive experience of the intervention	19
Preferred treatment features		Most helpful aspect of treatment program	
	Non-specific feature: group support	Nonspecific features of the program, including opportunities to receive peer support and a non-judgmental space for self-disclosure	19
	ACT-specific features	ACT-specific features of the intervention targeting mindfulness and parenting reactivity	5
Unexpected focus		Unexpected focus of ACT-PAC being primarily on parents’ experiences and behaviors and not on those of their anxious children	13
	Identified as unexpected impact	Focus on the parent experience seen as an unexpected of the intervention program	5
	Identified as intervention strength	Focus on parent experience perceived as helpful	8
Suggested improvements			
	Greater between-session accountability	Recommendations for increasing accountability from the group for between-session practice	3
	Additional resources	Suggestions for additional resources, particularly for opportunities to continue or extend engagement in skills practice	3
	Treatment Structure	Suggestions related to modifying the structure of group sessions	11
**Focus Group Feedback**			
Code		Definition	Number of Participating Caregivers That Reported Code Participating Caregivers (*n* = 7)
Positive features		Aspects of the ACT-PAC intervention found useful	7
	Non-specific feature: Group support and sharing common experiences	Group intervention offered an opportunity to connect to other caregivers struggling with similar parenting challenges	4
	ACT-specific content: Mindfulness	Mindfulness reported as among the more helpful features of the intervention	5
	ACT-specific content: Cognitive Defusion	Cognitive defusion reported as one of the most utilized skills and the easiest to generalize outside of session	4
Barriers to generalizing skills		Barriers to generalizing skills outside of treatment sessions	
	Limited accountability structure	Reports that accountability structure incentivizing practice not built into the treatment program	4
	Co-parenting challenges	Reports of challenges of implementing skills alongside a co-parent who did not attend the treatment sessions	5
	Forgetting and difficulty applying content between sessions	Reports of difficulty remembering the content between sessions, particularly due to lacking access to content material between the sessions	6
Future directions: Expanding target population		Reports that the ACT-PAC intervention would be beneficial for all parents, regardless of whether their children had an anxiety disorder	3

## Data Availability

The datasets presented in this article are not readily available because HIPPA. Requests to access the datasets should be directed to jrafteryhelmer@worcester.edu.

## Appendix A

### Appendix A.1. Individual Feedback Interview

What aspects of the group treatment program were most helpful to you?

What aspects of the group treatment program were least helpful?

How might we improve the group treatment program for future parents?

### Appendix A.2. Focus Group Questions

**Parenting Group—Therapist-Led Parent Training Sessions**
Let’s start by thinking about the parenting group meetings you attended weekly for 6 weeks.
1. What did you like the best about the parenting group meetings? Why?
2. What did you like the least about the parenting group meetings? Why? How would you recommend we improve the parenting group meetings?
There were several specific skills and practices that you did during the group meetings. These included mindfulness meditation practices, practices focused on seeing your thoughts as “just thoughts,” like the “three wishes” exercises, exercises to identify parenting values like “eighty year old you”, and self-care planning and practice exercises.
3. Which of the exercises or practices from the group did you like the most? Why?
4. Which of the exercises or practices from the group did you like the least? Why? How would you recommend we improve these exercises?
The group was scheduled weekly, on Tuesday nights, for 6 weeks.
5. Did the weekly schedule work for you, or would you have preferred to have met for 2 longer sessions on weekend days, like coming for 2 Saturday meetings for 3 h each?
Finally, we would like you to comment on your experience of the group leaders. Please note that, depending on the group cycle you attended, you may have had different leaders than others here today. That said, you do not need to specifically identify the leaders the group you participated in.
6. What did you like or dislike about the parenting group leaders? Why? How might the leaders do better?

**Parenting Group—Individual Practice At Home**
Think now about the exercises you worked on practicing at home. These included mindfulness practice, defusion practice (recognizing thoughts as thoughts), and practicing ‘towards moves’ which were commitments to act according to parenting values, in general and in the context of your child’s anxiety.
1. Were you able to do home practice? If yes, how many times a week were you able to find time to practice?
2. What were the major barriers to home practice of skills? How might the program address these barriers to home practice?
3. What did you like the best about practicing at home? Why?
4. What did you like the least? Why? How would you suggest we improve the at home practice for other parents?
5. When you did practice, what were the factors that helped you complete and benefit from that home practice?

**Home Use of Program Skills During and After Group Completion**
1. During the time you were in the group, did you feel you were able to use the skills learned in the group while you were interacting with your anxious child? If yes, how? If not, what do you think got in the way of this?
2. Which skills from the group, if any, do you still practice in your daily life and your interactions with your child? Why?
3. What changes would you recommend we make to this program to make group-learned skills more applicable to “real life” at home?
4. What do you think might be helpful in terms of **sustaining** the continued use of the group-learned skills, even after the group is over?

**General Suggestions**
1. Overall, how could we make the parenting group more useful or helpful for other parents of anxious children?
2. Would you recommend our group to other parents of anxious children? Why or why not?
